# Comprehensive evaluation of non-coding RNA-mediated autophagy regulation in myocardial ischemia-reperfusion injury

**DOI:** 10.3389/fphar.2025.1581341

**Published:** 2025-04-25

**Authors:** Zhongyang Song, Chang Suo, Yongqi Liu, Ling Jin, Xiaodong Xie, Jian Liu, Bo Yu, Yanzhen Wang, Zhiming Zhang, Dingxiong Xie

**Affiliations:** ^1^ Department of Oncology, Affiliated Hospital of Gansu University of Traditional Chinese Medicine, Lanzhou, Gansu, China; ^2^ Gansu Institute of Cardiovascular Diseases, Lanzhou, Gansu, China; ^3^ School of Pharmacy, Fujian Medical University, Fuzhou, Fujian, China; ^4^ Key Laboratory of Dunhuang Medicine and Transformation of Ministry of Education, Gansu University of Traditional Chinese Medicine, Lanzhou, Gansu, China; ^5^ Longyao Industry Innovation Institute, Gansu University of Traditional Chinese Medicine, Lanzhou, Gansu, China; ^6^ Institute of Genetics, School of Basic Medicine, Lanzhou University, Lanzhou, China; ^7^ Department of Critical Care Medicine, Maternal and Child Healthcare Hospital of Gansu Province, Lanzhou, China; ^8^ Department of General Surgery, The Second People’s Hospital of Lanzhou City, Lanzhou, China; ^9^ Department of Oncology, Gansu Provincial Hospital of Traditional Chinese Medicine, Lanzhou, Gansu, China

**Keywords:** myocardial ischemia-reperfusion injury, non-coding RNA, autophagy, research progress, molecular mechanism

## Abstract

Ischemic heart disease remains a major global health challenge, with myocardial ischemia-reperfusion injury (MIRI) being one of its most common and severe pathophysiological complications. The pathogenesis of MIRI is multifaceted, involving oxidative stress, inflammatory responses, apoptotic pathways, and autophagic regulation. Notably, autophagy exerts a dual regulatory effect, where maintaining optimal autophagic flux is essential for cardiac homeostasis. Emerging evidence underscores the crucial role of non-coding RNAs (ncRNAs) in modulating these pathological processes. In particular, long non-coding RNAs (lncRNAs), microRNAs (miRNAs), and circular RNAs (circRNAs) have been identified as key regulators of autophagy-mediated MIRI progression through complex molecular networks. This review provides a systematic analysis of the molecular pathways through which ncRNAs influence MIRI pathogenesis, with a specific focus on their autophagy-regulatory mechanisms. These insights may enhance our understanding of MIRI pathobiology and facilitate the development of novel therapeutic strategies.

## 1 Introduction

Acute myocardial infarction (AMI) is a severe cardiovascular condition caused by the occlusion of coronary arteries, which supply blood to the myocardium. It remains one of the leading causes of mortality worldwide (Zhu et al., 2024; Feng et al., 2024; Mastoor et al., 2025). Prompt reperfusion therapy is the cornerstone of AMI treatment and is essential for preserving myocardial integrity. However, some patients experience myocardial ischemia-reperfusion injury (MIRI) following revascularization (Gao et al., 2024; Jin et al., 2024; Tsurusaki and Kizana, 2024; Zhuang et al., 2024). This pathological process induces cardiomyocyte apoptosis, myocardial damage, the no-reflow phenomenon, and microvascular endothelial dysfunction, ultimately leading to complications such as arrhythmia, heart failure, and cardiogenic shock, which significantly impact patient prognosis (Taha and Shaker, 2021). The pathogenesis of MIRI is highly complex and involves multiple mechanisms, including oxidative stress, apoptosis, autophagy, ferroptosis, intracellular calcium overload, energy metabolism disorders, endoplasmic reticulum stress, and necroptosis (Xu et al., 2020; Tsai et al., 2021; Yang et al., 2021). Current therapeutic strategies focus on improving myocardial perfusion, reducing infarct size, and restoring myocardial function. Pharmacological interventions for MIRI primarily include angiotensin receptor antagonists, adenosine receptor agonists, statins, and magnesium-based agents. However, their clinical application remains limited, necessitating the exploration of novel therapeutic strategies and molecular targets (Xing et al., 2022). Consequently, understanding the pathogenesis of MIRI and identifying new pharmacological approaches to enhance myocardial perfusion have become pressing challenges in cardiovascular research.

Non-coding RNAs (ncRNAs) are a class of RNA molecules in eukaryotic cells that do not encode proteins. Studies indicate that approximately 98% of the human genome consists of ncRNAs, including long non-coding RNAs (lncRNAs), microRNAs (miRNAs), and circular RNAs (circRNAs) (Zhang et al., 2020). Growing evidence suggests that ncRNAs are significantly dysregulated in ischemic heart disease, highlighting their critical role in MIRI pathogenesis (Wadley et al., 2019; Goina et al., 2024; Yaghoobi et al., 2024; Makkos et al., 2021). Autophagy, a conserved catabolic process, maintains cellular homeostasis and survival. Moderate autophagy provides an adaptive response to hypoxic-ischemic injury in cardiomyocytes and exerts a protective effect. However, excessive autophagy may exacerbate ischemic heart disease progression (Chen G. et al., 2021; Wang et al., 2018; Aljakna Khan and Sabatasso, 2025; Yuan et al., 2025; Yang et al., 2025). Recent research has underscored the pivotal role of ncRNAs in cardiovascular diseases, particularly in regulating autophagy in cardiomyocytes (Sun et al., 2018). Therefore, elucidating the molecular mechanisms by which ncRNAs modulate autophagy in MIRI is crucial for developing targeted therapies. This review focuses on ncRNAs involved in MIRI through autophagy regulation in cardiomyocytes, aiming to provide novel therapeutic targets for MIRI prevention and treatment.

## 2 Autophagy

The molecular mechanisms of autophagy were first elucidated in the early 1990s by Prof. Yoshinori Ohsumi and his team, who identified autophagy-related genes in yeast (Ohsumi, 2014). Since then, the field of autophagy research has advanced significantly. Autophagy is categorized into three distinct types: macroautophagy, microautophagy, and chaperone-mediated autophagy, each differing in how cellular components are delivered to lysosomes for degradation. Among these, macroautophagy is the most widely expressed and well-characterized form in mammals (Ma et al., 2015). Autophagy is a crucial metabolic process that degrades aged or damaged proteins and organelles, breaking them down into amino acids and fatty acids for energy production and cellular recycling (Cătană et al., 2018). This process is particularly activated under nutrient deprivation or metabolic stress, playing a fundamental role in maintaining tissue function and dynamic homeostasis (Wu et al., 2021).

**FIGURE 1:**
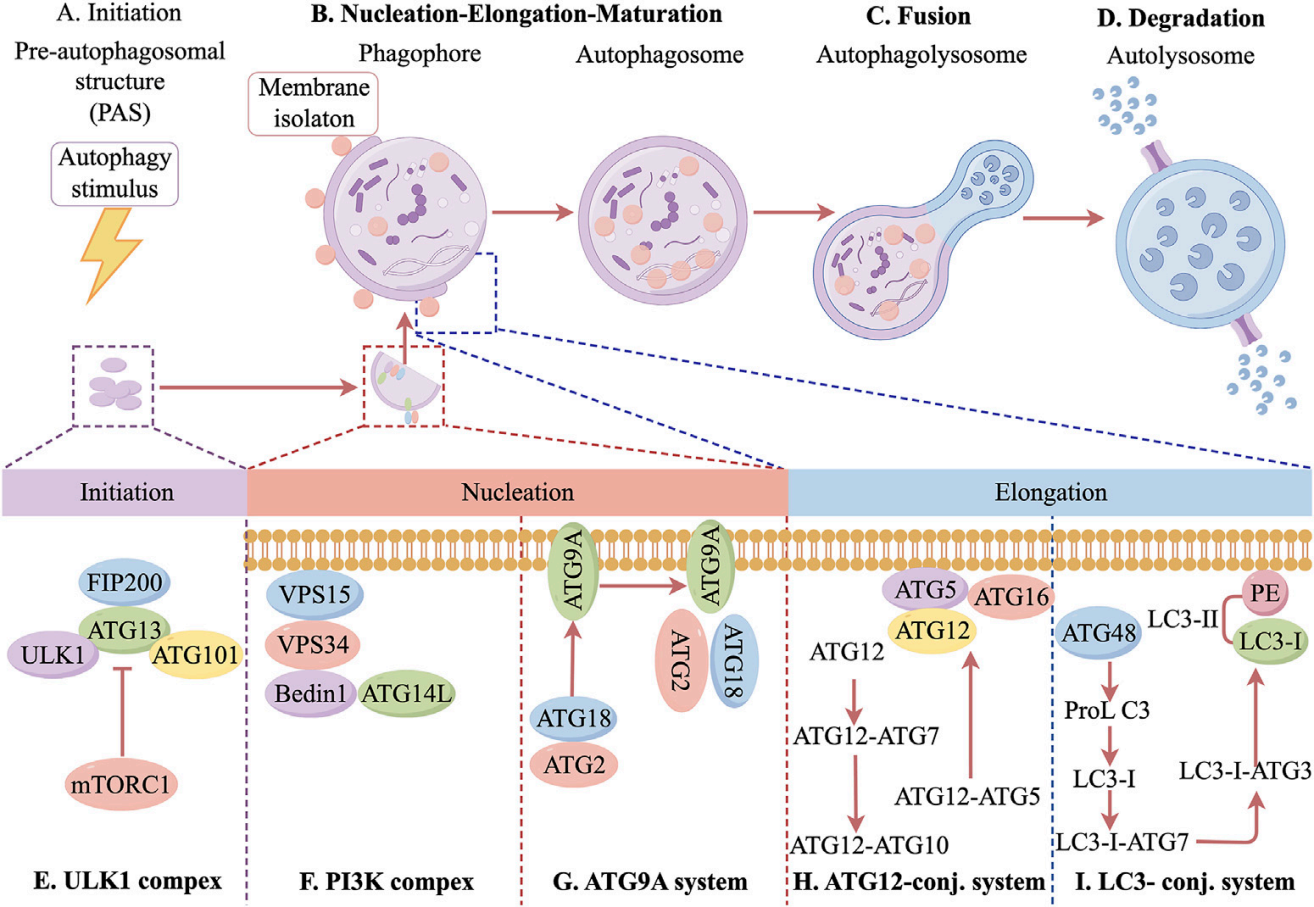
Molecular mechanisms of autophagy regulation.

As a major intracellular degradation and recycling system, autophagy is essential for cellular viability and homeostasis (Zhao et al., 2021; Ma et al., 2025). The autophagic process consists of several key stages, including autophagy initiation, autophagosome nucleation and maturation, fusion of autophagosomes with lysosomes, and autophagosome degradation (Hansen et al., 2018). Autophagy initiation is tightly regulated by multiple proteins and signaling pathways. Under stress conditions such as nutrient deprivation, infection, or inflammation, adenosine monophosphate-activated protein kinase (AMPK) is activated, while the mechanistic target of rapamycin complex 1 is inhibited. This inhibition promotes the UNC-51-like kinase 1 (ULK1) complex, which plays a key role in autophagy initiation. Additionally, the class III phosphatidylinositol 3-kinase (PI3K) complex, particularly PIK3C3/Vps34, is closely associated with this process. One of the key steps in autophagy regulation is the formation of autophagosomes, which requires activation of the PI3K complex. This activation stimulates Vps34 lipid kinase in the Beclin-1 complex, leading to the production of phosphatidylinositol 3-phosphate (PI3P), a crucial signaling molecule for autophagosome formation. PI3P recruits downstream autophagy-related (Atg) proteins, driving autophagosome assembly and expansion. Specifically, Atg proteins form the Atg5-Atg12-Atg16L1 complex under the guidance of PI3P. As the autophagosomal membrane extends, it encloses cytoplasmic material, eventually forming a mature autophagosome (Fujioka and Noda, 2025). The maturation and degradation of autophagosomes are tightly regulated by various factors. At the autophagosome membrane, the Atg12-Atg5-Atg16L1 complex facilitates the conversion of microtubule-associated protein 1 light chain 3 (LC3) into its active form. LC3 is a key autophagosome marker and plays a crucial role in autophagosome formation. The phosphatidylethanolamine/LC3 system converts LC3 from its inactive, water-soluble LC3-I form to the lipid-bound LC3-II form, which integrates into the autophagosome membrane and mediates autophagic activity (Maeyashiki et al., 2017). Another critical autophagy-related protein is ubiquitin-binding protein p62, which serves as a marker of autophagic capacity. Through its LC3-interacting region domain, p62 binds directly to LC3, mediating selective autophagy and facilitating the degradation of damaged proteins via the ubiquitin-proteasome pathway (Lin et al., 2013). Once mature, autophagosomes fuse with lysosomes to form autolysosomes, where degradation occurs. Within these complexes, lysosomal acid hydrolases break down encapsulated cellular components, releasing degradation products such as amino acids, proteins, and carbohydrates. These molecules are subsequently recycled for biosynthesis or utilized as an energy source, thereby contributing to intracellular homeostasis (Bednarczyk et al., 2024). In conclusion, autophagy is a highly regulated and complex biological process essential for cellular homeostasis. It is orchestrated by a network of proteins and signaling pathways, ensuring precise control over cellular degradation and recycling. Disruptions in autophagy can lead to cellular instability and contribute to the onset of various pathological conditions. The specific molecular mechanisms are shown in Figure 1. Therefore, a deeper understanding of autophagy regulation may provide new insights into disease mechanisms and potential therapeutic interventions.

## 3 Autophagy and MIRI

The pathogenesis of MIRI is highly complex and involves multiple physiological processes, including calcium ion overload, oxidative stress, inflammatory responses, abnormal energy metabolism, endoplasmic reticulum stress, mitochondrial dysfunction, autophagic dysregulation, and oxygen radical bursts (Bader and Winklhofer, 2020). Among these, autophagy plays a critical role in maintaining cellular homeostasis by degrading misfolded or dysfunctional proteins and damaged organelles, thereby stabilizing the intracellular environment. During myocardial ischemia, autophagy is activated to remove damaged cellular components, thereby mitigating oxidative stress and inflammatory responses, which in turn protects myocardial function. However, in the reperfusion phase, excessive autophagic activity continues to degrade normal organelles and mitochondria, leading to cardiomyocyte damage or even cell death (Mokhtari and Badalzadeh, 2021; Paradies et al., 2018). Regulating autophagy at an optimal level can attenuate oxidative stress and vascular endothelial injury, reduce cardiomyocyte apoptosis, and ultimately improve MIRI outcomes (Tang L. et al., 2021). Consequently, modulating autophagy during myocardial ischemia-reperfusion (I/R) has emerged as a key therapeutic strategy for MIRI management.

In the early stage of myocardial ischemia-reperfusion, blood supply to cardiomyocytes is restricted, leading to decreased ATP production and an imbalance between oxygen supply and energy demand. AMPK, a key autophagy initiator in myocardial ischemia, is activated in response to ATP depletion. Activated AMPK directly phosphorylates and activates ULK1, thereby triggering autophagy to protect cardiomyocytes from ischemic injury (Lin et al., 2018; Shen et al., 2024). Additionally, hypoxia-inducible factor 1-alpha (HIF-1α), a major regulator of cellular responses to hypoxia, plays a significant role in autophagy activation across various cell types. Under ischemic and hypoxic conditions, HIF-1α is upregulated in cardiomyocytes, potentially exerting a protective effect by modulating autophagy (Zhou et al., 2017). These findings suggest that autophagy is a protective response during the ischemic phase, activated in response to energy crises and oxidative stress, to preserve cardiomyocyte structure and function, thereby safeguarding cardiac physiology. Autophagy also plays a crucial role during myocardial reperfusion as an endogenous repair mechanism for ischemic myocardial injury. However, during reperfusion, excessive autophagic activation leads to cardiomyocyte autophagic death and exacerbates tissue damage (Catrysse and van Loo, 2017). While moderate autophagic activity is essential for cardiomyocyte survival during hypoxia-reoxygenation, excessive autophagy disrupts cellular homeostasis and contributes to further myocardial damage (Mokhtari and Badalzadeh, 2021). Therefore, understanding the dynamic regulation of autophagy during reperfusion is critical for developing therapeutic strategies aimed at minimizing myocardial injury and optimizing cardiomyocyte survival following ischemia-reperfusion.

## 4 Autophagy is involved in MIRI-related pathways

### 4.1 PI3K/Akt/mTOR pathway

The PI3K/Akt/mTOR signaling pathway plays a crucial role in autophagy regulation and cardiomyocyte survival during MIRI. Ginsenoside Rb1, a bioactive compound from Panax ginseng, has demonstrated cardioprotective effects. The addition of the autophagy inhibitor 3-methyladenine enhances the protective effects of ginsenoside Rb1 on H9c2 cardiomyocytes during hypoxia/reoxygenation (H/R) injury. This suggests that ginsenoside Rb1 exerts its protective function by inhibiting cardiomyocyte autophagy through the PI3K/Akt/mTOR signaling pathway, making it a potential therapeutic agent for MIRI (Qin et al., 2021). Similarly, acacetin, an active compound found in Ziziphus jujuba (jujube nut), has been shown to protect against MIRI. Acacetin treatment induces a dose-dependent upregulation of autophagy markers, including LC3-II, Beclin-1, and p62, indicating enhanced autophagic activity. This protective effect is attributed to PI3K/Akt/mTOR pathway activation, which promotes autophagy, inhibits excessive cell metabolism, and facilitates cell proliferation. These findings suggest that modulation of the PI3K/Akt/mTOR signaling pathway may provide a novel therapeutic strategy for reducing MIRI-induced cardiac damage (Liu et al., 2021).

### 4.2 AMPK/mTOR pathway

The AMPK/mTOR signaling pathway plays a crucial role in autophagy regulation and cardiomyocyte survival during MIRI. This pathway is involved in energy homeostasis and cellular stress responses, making it a key target for cardioprotective interventions. Cordycepin, a nucleoside antibiotic extracted from the traditional Chinese medicinal fungus Cordyceps sinensis, has demonstrated cardioprotective effects, including anti-inflammatory, antioxidant, and anti-differentiation properties. A study by Xu et al. (2022) found that cordycepin significantly reduced apoptosis, decreased infarct size, and improved MIRI outcomes in mice, while also enhancing autophagy. Further investigation revealed that the cardioprotective effects of cordycepin are mediated through autophagy activation via the AMPK/mTOR signaling pathway, highlighting its potential therapeutic application in MIRI treatment. Similarly, geniposide, an active compound extracted from Gardenia jasminoides, exhibits broad pharmacological effects. Research (Luo et al., 2020) suggests that geniposide exerts a protective effect against MIRI by modulating autophagy. In MIRI models, geniposide intervention led to a reduction in Beclin-1 levels, suggesting that it inhibits excessive autophagy, potentially through activation of the AMPK/mTOR signaling pathway. These findings indicate that targeting AMPK/mTOR-mediated autophagy regulation may serve as an effective strategy for mitigating MIRI-induced myocardial damage.

### 4.3 SIRT3 pathway

Sirtuin 3 (SIRT3) is a key member of the histone deacetylase family, known for its role in mitochondrial function, oxidative stress regulation, and autophagy modulation. Studies have shown that selegiline, a monoamine oxidase inhibitor, significantly attenuates H/R injury-induced autophagy overactivation in cardiomyocytes, while simultaneously upregulating SIRT3 expression. Notably, SIRT3 knockdown reduces the protective effects of selegiline against H/R-induced autophagy and cardiomyocyte injury, suggesting that selegiline exerts its cardioprotective effects by modulating SIRT3 and autophagy (Yang et al., 2022). Benzo[a]pyrene (BaP), a widely studied polycyclic aromatic hydrocarbon, has been associated with an increased risk of cardiovascular disease. Research by (Huang et al., 2023) demonstrated that BaP activates the p53-protein-interacting protein 3 (BNIP3) pathway via the aryl hydrocarbon receptor, thereby reducing autophagic vesicle clearance. This finding suggests that the p53-BNIP3 pathway, which plays a crucial role in autophagy regulation, may serve as a potential therapeutic target for BaP-induced MIRI.

### 4.4 BNIP3 pathway

BCL2/adenovirus E1B 19 kDa BNIP3 is a pro-apoptotic protein and a member of the Bcl-2 family, which plays a crucial role in regulating autophagy-lysosomal fusion (Popov et al., 2023). BNIP3 is a target molecule of HIF-1α and can be upregulated under hypoxic or ischemic conditions, contributing to autophagy modulation. A study by Zhang et al. demonstrated that in H9c2 cardiomyocytes exposed to H/R conditions, HIF-1α activation synchronously regulated the BNIP3 pathway, leading to increased BNIP3 expression (Zhang et al., 2019). This upregulation enhanced autophagic activity in H9c2 cardiomyocytes, thereby alleviating MIRI-related cardiac damage. These findings suggest that targeting the BNIP3 pathway may serve as a potential therapeutic strategy for modulating autophagy in MIRI.

## 5 Non-coding RNA regulation of autophagy in MIRI

### 5.1 MiRNAs

#### 5.1.1 Overview of miRNAs

MiRNAs are a class of small endogenous ncRNAs present in eukaryotic cells, typically consisting of 18–24 nucleotides. They regulate post-transcriptional gene expression by binding to the 3′ untranslated region (3′UTR) of target messenger RNAs (mRNAs), leading to translational inhibition or mRNA degradation (Jámbor et al., 2019). Most miRNA genes are located in intergenic regions and form primary, independent transcription units. In the presence of RNA polymerase II, miRNA genes are transcribed into primary miRNA transcripts (pri-miRNAs), which are thousands of nucleotides long. These pri-miRNAs undergo processing by RNAse III enzyme Drosha, RNA-binding proteins, and DiGeorge syndrome-associated protein 8, leading to the formation of pre-miRNAs with a hairpin structure (∼70 nucleotides in length). Pre-miRNAs are then transported from the nucleus to the cytoplasm by Exportin-5. In the cytoplasm, Dicer, an RNAse III enzyme, further processes pre-miRNAs by cleaving their hairpin loops, generating miRNA duplexes. These duplexes are subsequently unwound by helicase enzymes, resulting in two strands: The guide strand (mature miRNA), which interacts with the Argonaute protein to form the RNA-induced silencing complex and the passenger strand, which is typically degraded as a substrate for RISC. Once incorporated into the RISC complex, miRNAs bind to the 3′UTR of target genes, acting as negative regulators of gene expression through either translation inhibition or mRNA degradation. Each miRNA can regulate multiple target genes, while individual genes may also be regulated by multiple miRNAs. Although miRNAs account for approximately 4% of human genes, they are involved in the regulation of nearly 30% of the human genome (Dosil et al., 2022). Studies indicate that cell proliferation, differentiation, and apoptosis are among the biological processes significantly influenced by miRNAs (Galagali and Kim, 2020).

#### 5.1.2 MiRNAs promote autophagy to attenuate MIRI

Several studies have demonstrated that certain miRNAs play a crucial role in mitigating MIRI by promoting autophagy, which reduces cardiomyocyte death and protects against ischemic-hypoxic damage. During MIRI, the inhibition of miR-490-3p or the overexpression of ATG4B has been shown to enhance LC3-II expression, increase autophagosome formation, suppress p62 expression, and reduce infarct size (Wu et al., 2021). These findings suggest that silencing miR-490-3p expression leads to ATG4B upregulation, thereby promoting autophagy and protecting cardiomyocytes from reperfusion injury. Similarly, miR-497 expression was significantly downregulated in myocardial infarcted hearts and in cultured neonatal rat cardiomyocytes. Both *in vivo* and *in vitro* studies demonstrated that low miR-497 expression increased autophagic flux, suggesting that miR-497 inhibition attenuates MIRI by enhancing autophagy (Li et al., 2015). Additionally, the miR-30 family member, miR-30e-3p, was significantly downregulated in a cardiomyocyte model of ischemic-hypoxic rats. Overexpression of miR-30e-3p led to a notable increase in LC3-II levels and a significant decrease in p62 and Egr-1 expression, effectively reducing ischemic-hypoxia-induced myocardial injury. These findings indicate that miR-30e-3p overexpression suppresses Egr-1 expression, enhances autophagy, and attenuates cardiomyocyte injury (Su et al., 2020). Furthermore, miR-122 was significantly upregulated in hypoxic H9c2 cardiomyocytes. Overexpression of miR-122 inhibited the activation of the PI3K/Akt autophagy-associated pathway, leading to increased hypoxia-induced apoptosis in H9c2 cardiomyocytes (Zhang et al., 2017). These results suggest that inhibiting miR-122 expression regulates the PTEN/PI3K/Akt signaling pathway, thereby promoting autophagy, enhancing cardiomyocyte viability, and protecting against hypoxia-induced injury.

#### 5.1.3 MiRNAs inhibit autophagy to attenuate MIRI

H/R injury is a major contributor to MIRI, often leading to excessive autophagy in cardiomyocytes, which can exacerbate cardiac tissue damage. Several miRNAs have been identified as negative regulators of autophagy, helping to protect cardiomyocytes from H/R-induced injury. miR-16-5p is highly expressed in H/R-treated AC16 cardiomyocytes, with significant increases in myocardial injury markers, including lactate dehydrogenase and creatine kinase isozymes. Knockdown of miR-16-5p significantly inhibited autophagic flux in H/R-treated AC16 cells, while silencing of protein tyrosine phosphatase non-receptor type 4 (PTPN4) reversed this effect. These findings suggest that miR-16-5p alleviates H/R-induced myocardial injury by inhibiting autophagy through the negative regulation of PTPN4 expression (Cao et al., 2021). Similarly, miR-451-3p was significantly downregulated in a MIRI rat model. This downregulation promoted autophagy by activating microtubule-associated protein 1 light chain 3 beta (MAP1LC3B), exacerbating myocardial injury. Restoring miR-451-3p expression was found to suppress autophagy, suggesting that miR-451-3p may serve as a novel molecular target for MIRI treatment (Lv et al., 2021a). Additionally, Zhang et al. demonstrated that miR-103a-3p exerts a protective role in MIRI by targeting Atg5, thereby inhibiting excessive autophagy (Zhang et al., 2019). These findings provide new insights into miRNA-based therapeutic strategies for MIRI. Moreover, HIF-1α, which is highly expressed in H/R-induced cardiomyocytes, plays a crucial role in autophagy induction across multiple cell types. Increased expression of miR-590-3p inhibits excessive autophagy in cardiomyocytes by directly targeting HIF-1α, thereby alleviating MIRI (Gong et al., 2021). Further research has demonstrated that miR-101 is lowly expressed in both *ex vivo* and *in vivo* MIRI mouse models. Overexpression of miR-101 attenuates ischemia-reperfusion-induced myocardial injury by regulating autophagy through its target DNA damage-inducible transcript 4 (DDIT4) (Li Q. et al., 2020). Similarly, miR-34a expression was found to be reduced in rat cardiomyocytes and cardiac tissues after reperfusion. Overexpression of miR-34a decreased LC3-II and p62 expression in H/R-treated cardiomyocytes, indicating that miR-34a inhibits autophagy following reperfusion injury, thereby reducing myocardial damage (Shao et al., 2018). Finally, miR-210 has been reported to inhibit autophagy by upregulating p62 levels and decreasing the LC3-II/I ratio in hypoxic cardiomyocytes. These findings suggest that miR-210 suppresses autophagy to mitigate hypoxia-induced myocardial injury (Wu et al., 2021).

Accumulating evidence suggests that miRNAs play a crucial role in attenuating MIRI by regulating autophagy in cardiomyocytes through the AMPK/mTOR and Akt/mTOR pathways (Liu et al., 2017). In H_2_O_2_-induced H9c2 cells, oxidative stress reduced cell viability while increasing autophagic activity. Overexpression of miR-129-5p inhibited autophagy and alleviated H_2_O_2_-induced cellular injury. Further studies revealed that miR-129-5p suppresses autophagy by targeting ATG14 and activating the phosphorylation of the PI3K/Akt/mTOR pathway, thereby reducing oxidative stress-induced damage in cardiomyocytes (Zhang et al., 2018). Additionally, sirtuin 1 (SIRT1) is a key regulator of apoptosis and autophagy (Luo et al., 2019). Overexpression of miR-494 was found to downregulate SIRT1 expression while modulating the PI3K/Akt/mTOR signaling pathway, ultimately reducing both apoptosis and autophagy in cardiomyocytes, thus protecting against ischemia-reperfusion injury (Ning et al., 2020). Similarly, in ischemia-hypoxia-injured H9c2 cells, miR-21 expression was significantly reduced. Phosphatase and tensin homolog (PTEN), a well-known tumor suppressor, is a downstream target of miR-21. Upregulation of miR-21 leads to PTEN suppression, thereby activating the PI3K/Akt/mTOR pathway, inhibiting autophagy, and reducing cardiomyocyte death (Huang et al., 2017). Moreover, miR-223 was highly expressed in both human infarcted myocardial tissues and rat models of myocardial infarction. miR-223 protects cardiomyocytes from hypoxia-induced excessive autophagy by targeting the poly (ADP-ribose) polymerase 1 (PARP-1)-mediated Akt/mTOR pathway. These findings suggest that miR-223 may serve as a potential therapeutic target for MIRI (Liu C. Y. et al., 2018).

Exosomes are cell-derived nanovesicles that contain mRNAs, miRNAs, proteins, and lipids, playing a crucial role in regulating biological processes in target cells (Ma et al., 2021). Numerous studies have demonstrated that exosomal miRNAs contribute significantly to cardioprotective mechanisms (Nasser et al., 2021). It has been reported that miR-29c is highly expressed in bone marrow mesenchymal stem cell (MSC)-derived exosomes. Overexpression of miR-29c was found to protect the heart from cardiac reperfusion injury by reducing excessive autophagy through PTEN inhibition and AKT/mTOR pathway activation during myocardial reperfusion. Additionally, miR-29c directly targets and negatively regulates checkpoint kinase 2 (CHK2) expression, further contributing to cardioprotection (Li T. et al., 2020). Both *ex vivo* and *in vivo* studies have confirmed that exosomal miR-143-3p regulates autophagy via the CHK2-Beclin2 pathway, effectively reducing cell death, alleviating cardiomyocyte injury, and promoting cardiomyocyte repair (Chen H. Y. et al., 2021). These findings suggest that MSC-derived exosomal miR-143-3p may serve as a promising therapeutic strategy for reperfusion injury. Additionally, since ischemia-reperfusion leads to excessive autophagy, exosome-carried miR-30a inhibitor can suppress cardiomyocyte death in reperfusion-injured rats by reducing autophagic hyperactivation, which in turn diminishes infarct size and improves cardiac function (Xu et al., 2019).

#### 5.1.4 MiRNAs promote/inhibit autophagy to ameliorate MIRI

**FIGURE 2:**
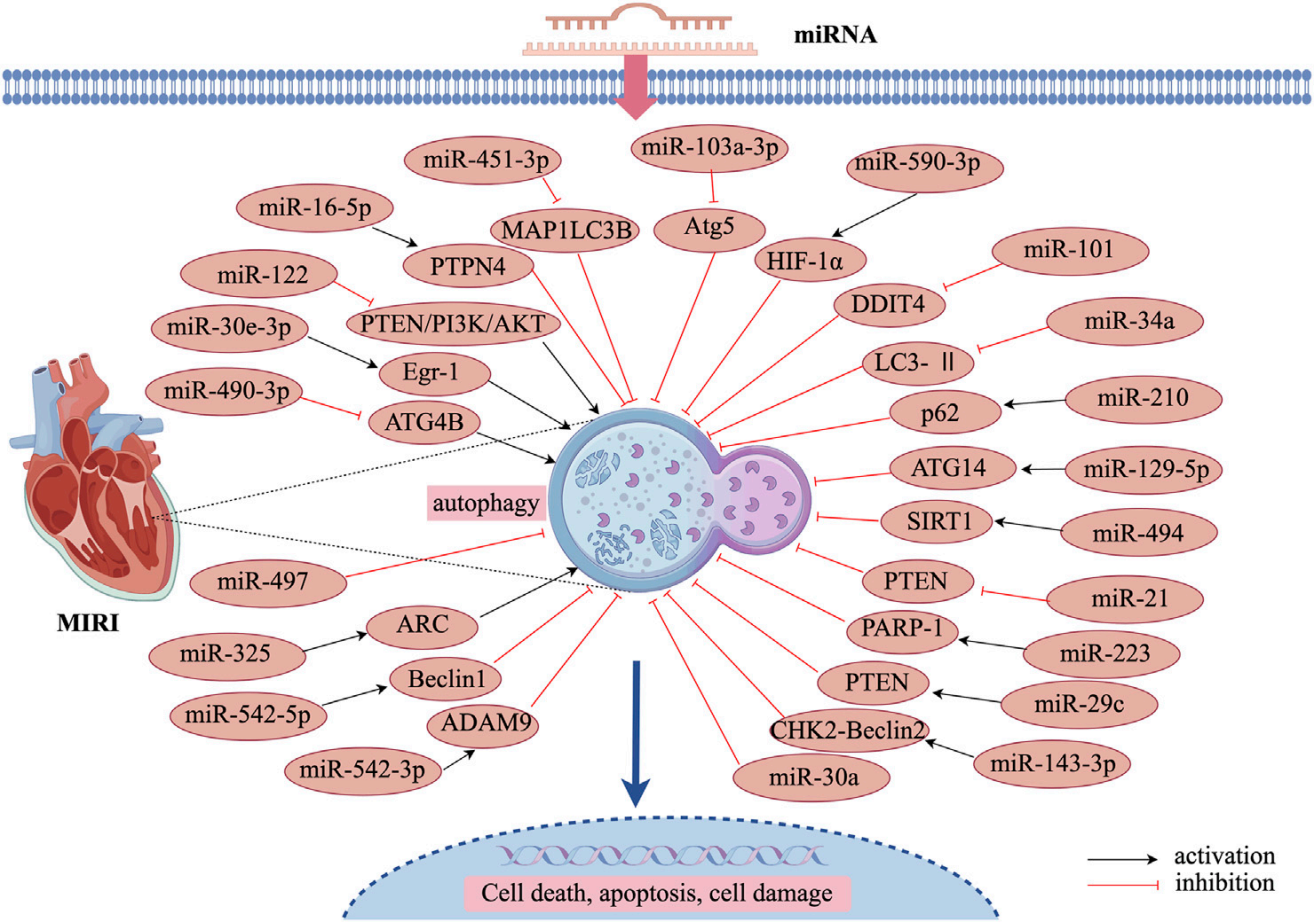
Mechanism of miRNA-regulated autophagy in MIRI.

Some miRNAs can either promote or inhibit autophagy, leading to exacerbation of myocardial reperfusion injury, which negatively impacts cardiac function. miR-542-5p expression and autophagy activation were significantly increased in H9c2 cells following H/R injury. Forced overexpression of miR-542-5p further aggravated H/R-induced myocardial injury, potentially by targeting the autophagy-related gene ATG7, thereby inhibiting autophagy and worsening cardiac injury (Wang et al., 2021). Furthermore, in a hypoxia/reperfusion cell model, reduced miR-542-3p expression was associated with impaired cell viability, increased apoptosis, and enhanced oxidative stress injury. In I/R mouse models, Beclin1 levels were elevated while p62 levels were reduced, indicating increased autophagic activity. However, following miR-542-3p overexpression, Beclin1 levels were downregulated and p62 levels were upregulated, leading to attenuation of cardiomyocyte injury (Ao et al., 2024). Another study demonstrated that miR-325 was upregulated in ischemia-reperfusion injury mouse cardiomyocytes. Overexpression of miR-325 was found to enhance autophagy, thereby expanding the myocardial infarction area. Additionally, E2F transcription factor 1 was shown to promote miR-325 expression, which in turn inhibited apoptosis repressor with caspase recruitment domain (ARC). Inhibition of ARC by miR-325 prevented ARC from suppressing autophagy, leading to autophagy overactivation and worsening MIRI (Bo et al., 2014). The specific regulatory mechanisms of these miRNAs in MIRI-related autophagy modulation are summarized in Figure 2 and Table 1.

**TABLE 1:** Role of miRNA-regulated autophagy in MIRI.

miRNA	Voice	Sample type	Target of action	Autophagy regulation	Mechanism of action	References
miR-490-3p	demote	H9c2 cardiomyocytes	ATG4B	Promoting autophagy	Reduction of myocardial infarct size	Wu et al. (2021)
miR-497	demote	Rat cardiomyocytes		Promoting autophagy	Reduces myocardial cell damage	Li et al. (2015)
miR-30e-3p	adjust upwards	Rat cardiomyocytes	Egr-1	Promoting autophagy	Reduces cardiomyocyte damage	Su et al. (2020)
miR-122	demote	H9c2 cardiomyocytes	PTEN/PI3K/AKT	Promoting autophagy	Enhancement of cardiomyocyte viability	Zhang et al. (2017)
miR-16-5p	adjust upwards	AC16 cardiomyocytes	PTPN4	Inhibition of autophagy	Reduces cardiomyocyte damage	Cao et al. (2021)
miR-451-3p	demote	Rat MIRI model	MAP1LC3B	Inhibition of autophagy	For myocardial ischemia-reperfusion Injuries play a protective role	Lv et al. (2021a)
miR-103a-3p	adjust upwards	H9c2 cardiomyocytes	Atg5	Inhibition of autophagy	Inhibition of hypoxia-reoxygenation induction cell death	Zhang et al. (2019)
miR-590-3p	adjust upwards	H9c2 cardiomyocytes	HIF-1α	Inhibition of autophagy	Inhibition of cardiomyocyte apoptosis	Gong et al. (2021)
miR-101	demote	H9c2 cardiomyocytes	DDIT4	Inhibition of autophagy	Reducing myocardial infarction-induced injury	Li Q. et al. (2020)
miR-34a	demote	Rat cardiomyocytes	LC3- II, p62	Inhibition of autophagy	Reduces myocardial damage	Shao et al. (2018)
miR-210	adjust upwards	H9c2 cardiomyocytes	p62, LC3-II/I ratio	Inhibition of autophagy	Reducing hypoxia-induced heart muscle damage	Wu et al. (2021)
miR-129-5p	adjust upwards	H9c2 cardiomyocytes	ATG14	Inhibition of autophagy	Reduces cellular damage	Zhang et al. (2018)
miR-494	adjust upwards	H9c2 cardiomyocyte and hypoxia mouse models	SIRT1	Inhibition of autophagy	Reduction of cardiomyocyte apoptosis	Ning et al. (2020)
miR-21	adjust upwards	H9c2 cardiomyocyte and hypoxia mouse models	PTEN	Inhibition of autophagy	Reduced cardiomyocyte death	Huang et al. (2017)
miR-223	adjust upwards	Rat cardiomyocytes	PARP-1	Inhibition of autophagy	Prevents hypoxia-induced cellular damage	Liu X. et al. (2018)
miR-29c	adjust upwards	Bone marrow mesenchymal stem cells and mice	PTEN	Inhibition of autophagy	Protecting the heart from oxidative stress	Li et al. (2020b)
miR-143-3p	adjust upwards	rats and H9c2 cardiomyocyte	CHK2-Beclin2	Inhibition of autophagy	Promote cardiomyocyte repair	Chen L. et al. (2021)
miR-30a	adjust upwards	Rat cardiomyocytes		Inhibition of autophagy	Reduction of infarct size, modification of charitable function	Xu et al. (2019)
miR-542-3p	adjust upwards	Mouse cardiomyocytes	ADAM9	Inhibition of autophagy	Reduces myocardial damage	Wang et al. (2021)
miR-542-5p	adjust upwards	H9c2 cardiomyocytes	Beclin1 and p62	Inhibition of autophagy	Aggravated myocardial damage	Ao et al. (2024)
miR-325	adjust upwards	Mouse cardiomyocytes	ARC	Promoting autophagy	Increased infarct size and myocardial damage	Bo et al. (2014)

### 5.2 LncRNA

#### 5.2.1 Overview of lncRNAs

LncRNAs are a class of RNA transcripts exceeding 200 nucleotides in length that do not encode proteins. In recent years, lncRNAs have gained significant attention, and they can be classified based on their genomic locations and functional roles into the following categories: sense-strand lncRNAs, antisense-strand lncRNAs, bidirectional lncRNAs, intergenic lncRNAs, enhancer-associated lncRNAs, and intronic lncRNAs (Herman et al., 2022). Compared to mRNAs, lncRNAs are generally shorter, contain fewer introns, and have lower expression levels. However, their expression is more tightly regulated than that of mRNAs, highlighting their functional significance (Quinn and Chang, 2016). LncRNAs exhibit diverse biological functions, including: (1) Cis-regulatory effects–Recruiting histone modification complexes to their transcription sites and interacting with them to modulate gene expression. (2) Trans-regulatory effects–Recruiting histone modification complexes to distant transcription sites, altering chromatin states and regulating distal gene expression. (3) RNA interaction and scaffolding–Regulating RNA activity through sequence complementation and providing a structural platform for protein interactions. (4) Transcriptional modulation - Enhancing or inhibiting regulator binding in non-coding regions to modulate gene expression. Micro-peptide encoding-Some lncRNAs encode functional micro-peptides, challenging the notion that they lack protein-coding potential (Batista and Chang, 2013; Ransohoff et al., 2018). Approximately 40% of lncRNAs exhibit brain-specific expression and are involved in neuronal growth, cell proliferation, migration, and apoptosis, processes that are closely associated with nervous system development and function (Zimmer-Bensch, 2019). These findings highlight the potential roles of lncRNAs not only in neurological processes but also in other pathophysiological conditions, including cardiovascular diseases such as MIRI.

#### 5.2.2 LncRNA-promoted autophagy attenuates MIRI

LncRNAs play a significant role in regulating various biological processes, including autophagy in both cardiac and non-cardiac cells. Recent studies have demonstrated that lncRNAs promote autophagy during MIRI, thereby protecting cardiomyocytes from ischemic damage. Lv et al. (2021b) reported that lncRNA H19 interacts directly with miR-143, functioning as a molecular sponge to suppress miR-143 expression. Since ATG7 is a target gene of miR-143, the competitive binding of lncRNA H19 to miR-143 leads to increased ATG7 expression, thereby enhancing autophagy. These findings suggest that lncRNA H19 alleviates MIRI by promoting autophagy via the H19/miR-143/ATG7 axis. Endoplasmic reticulum (ER) stress plays a critical role in MIRI pathogenesis. Research has shown that lncRNA Dancr regulates ER stress in cardiomyocytes. Tunicamycin treatment induces autophagy activation, characterized by increased Beclin-1 levels, an elevated LC3-II/I ratio, and decreased p62 expression. Overexpression of lncRNA Dancr further enhances autophagy while simultaneously reducing miR-6324 expression. Notably, miR-6324 overexpression counteracts the autophagy-promoting effects of lncRNA Dancr, suggesting that lncRNA Dancr enhances autophagy via competitive binding to miR-6324, thereby restoring ER homeostasis and mitigating MIRI (Li et al., 2021a). Furthermore, lncRNA 2810403D21Rik (also known as Mirf) was found to be significantly upregulated in H_2_O_2_-treated cardiomyocytes and in cardiac tissues of a myocardial infarction mouse model. In contrast, miR-26a, which functions as its competing endogenous RNA (ceRNA), was significantly downregulated in both cardiomyocytes and ischemic myocardial tissues. Experimental studies revealed that silencing lncRNA 2810403D21Rik/Mirf or overexpressing miR-26a led to reduced autophagy levels, smaller infarct size, and improved cardiac function. Further investigation showed that miR-26a overexpression activates cardiomyocyte autophagy by directly targeting Usp15 (Liang et al., 2020). These findings suggest that lncRNA 2810403D21Rik/Mirf exerts its cardioprotective effects by competitively binding to miR-26a and regulating Usp15 expression, thereby enhancing autophagy and protecting cardiomyocytes from ischemia-reperfusion injury.

#### 5.2.3 LncRNA inhibition of autophagy attenuates MIRI

MIRI often leads to excessive autophagy activation in cardiomyocytes, which can further exacerbate cardiac damage. Certain lncRNAs help mitigate MIRI by inhibiting overactive autophagy, thereby maintaining cellular homeostasis. These lncRNAs regulate autophagy-related genes such as Beclin-1, Atg5, Atg7, and Atg12, which are essential in controlling autophagy flux. Li et al. (2021b) reported that lncRNA KCNQ1OT1 was overexpressed in myocardial infarction patients, reperfusion injury mice, and H/R-induced cardiomyocyte models. A luciferase reporter assay confirmed that lncRNA KCNQ1OT1 suppressed miR-26a-5p expression, which in turn inhibited ATG12 expression. Notably, KCNQ1OT1 upregulation inhibited autophagy, thereby protecting cardiomyocytes from ischemia-reperfusion injury via the KCNQ1OT1/miR-26a-5p/ATG12 axis. Additionally, ATG7 is a key autophagy-promoting gene that plays a crucial role in reperfusion-induced myocardial injury. Research has shown that miR-188-3p inhibits autophagy and myocardial infarction by directly targeting ATG7. However, lncRNA autophagy-promoting factor (APF) binds to miR-188-3p, reducing its activity (Wang et al., 2015). These findings indicate that lncRNA APF protects cardiomyocytes by regulating the APF/miR-188-3p/ATG7 axis to suppress autophagy and myocardial infarction. Furthermore, lncRNA PVT1 was upregulated in H/R-treated human AC16 cardiomyocytes. Knockdown of PVT1 was found to block H/R-induced injury, while also reducing excessive autophagy. This was confirmed by decreased LC3-II and Beclin-1 expression, increased p62 expression, and reduced autophagic vesicle accumulation. As PVT1 functions as a ceRNA for miR-186, its knockdown protected AC16 cardiomyocytes from hypoxia/reoxygenation-induced injury through the miR-186/Beclin-1 axis (Ouyang et al., 2020). Additionally, lncRNA prostate androgen-regulated transcript 1 (PART1), a newly identified lncRNA, was found to elevate LC3, Beclin-1 expression, and the LC3-II/I ratio, while decreasing p62 levels in H/R-mediated cardiomyocyte injury. However, overexpression of PART1 resulted in reduced LC3 and Beclin-1 expression, a lower LC3-II/I ratio, and elevated p62 levels, indicating an autophagy-suppressive effect. Interestingly, miR-302a-3p upregulation counteracted PART1’s autophagy-regulating effects, suggesting that PART1 inhibits H/R-induced autophagy by negatively regulating miR-302a-3p (Zeng et al., 2024).

Several lncRNAs, including X-inactive specific transcript (XIST), taurine upregulated gene 1 (TUG1), and testis-specific transcript, Y-linked 15 (TTTY15), have been identified as key regulators of autophagy in MIRI. These lncRNAs have been validated and found to be highly expressed in *ex vivo* reperfusion injury models, highlighting their potential translational significance in clinical applications. Li et al. (2019) reported that lncRNA XIST was upregulated in H/R-treated cardiomyocytes. Silencing XIST inhibited autophagy, which in turn increased cell viability. Furthermore, miR-133a overexpression mitigated H/R-induced cellular damage and autophagy by regulating suppressor of cytokine signaling 2 (SOCS2). These findings suggest that low XIST expression confers cardioprotection during myocardial ischemia-reperfusion by inhibiting autophagy and modulating the miR-133a/SOCS2 axis. Similar to XIST, lncRNA TUG1 was upregulated, while miR-142-3p was downregulated in *ex vivo* models of myocardial reperfusion injury. Studies demonstrated that TUG1 silencing and miR-142-3p overexpression suppressed cardiomyocyte apoptosis and autophagy, with high-mobility group box 1 (HMGB1) and Rac1 playing key roles in mediating TUG1-induced apoptosis and autophagy (Su et al., 2019). These results indicate that TUG1 contributes to myocardial injury and acute myocardial infarction progression by targeting miR-142-3p and upregulating HMGB1 and Rac1. Likewise, lncRNA TTTY15 was highly expressed in both *in vivo* and *ex vivo* myocardial reperfusion injury models. TTTY15 regulates miR-374a-5p expression, which h in turn influences forkhead box O1 (FOXO1) expression and autophagy in cardiomyocytes during reperfusion injury (Chen L. et al., 2021). These findings suggest that TTTY15 plays a crucial role in modulating autophagy and apoptosis in ischemia-reperfusion injury.

Cardiac myosin is a nuclear protein specifically expressed in the myocardium, and its knockdown has been shown to inhibit autophagy and mitigate ischemia-induced myocardial injury. The tumor suppressor protein p53 plays a key role in upregulating cardiac myosin expression, thereby activating autophagy. However, excessive autophagy activation via the p53–cardiac myosin signaling axis leads to cardiomyocyte death and worsened ischemic damage. Studies have demonstrated that lncRNA CAIF directly interacts with p53, preventing its binding to the cardiac myosin promoter, which subsequently reduces cardiac myosin expression (Liu X. et al., 2018). This mechanism inhibits autophagic cell death in MIRI and reduces infarct size, suggesting that lncRNA CAIF confers cardioprotection via the CAIF–p53–cardiac myosin signaling axis. LINC00174, an exosome-derived lncRNA, was found to be downregulated in reperfusion-injured mice. Experimental data indicate that LINC00174 alleviates ischemia-reperfusion-induced myocardial injury by inhibiting autophagy, apoptosis, and vacuole formation in cardiomyocytes. Mechanistically, LINC00174 directly regulates serine/arginine-rich splicing factor 1 (SRSF1) expression, which in turn suppresses p53 and cardiac troponin levels in cardiomyocytes (Su et al., 2021). These findings suggest that the LINC00174–SRSF1–p53–cardiac myosin pathway may serve as a potential therapeutic target for attenuating excessive autophagy following ischemia-reperfusion injury, thereby improving cardiac function. lncRNA UCA1 is another autophagy-regulating lncRNA that is downregulated in H/R-treated cardiomyocytes and myocardial tissues. UCA1 inhibits excessive autophagy in cardiomyocytes by acting as a molecular sponge for miR-143, thereby modulating Bcl-2 expression and protecting cardiac microvascular endothelial cells from H/R-induced injury (Diao and Zhang, 2021). Additionally, ischemia-reperfusion injury induces excessive autophagy in cardiomyocytes, and UCA1 is downregulated in ischemia-reperfusion heart tissues. Research suggests that UCA1 directly targets miR-128, which in turn regulates heat shock protein 70 (HSP70) expression. Interestingly, morphine treatment significantly inhibits excessive autophagy by modulating the UCA1/miR-128/HSP70 axis, thereby reducing cardiomyocyte autophagy, minimizing cell death, and decreasing infarct size (Chen et al., 2018). These findings highlight the potential therapeutic role of UCA1 in alleviating MIRI through autophagy suppression.

#### 5.2.4 LncRNA promotion/inhibition of autophagy attenuates MIRI

Certain lncRNAs are upregulated following MIRI and exacerbate cardiac damage by suppressing miRNA expression and enhancing autophagy-related target gene expression. CHRF, an lncRNA that is upregulated in MIRI models, has been shown to negatively regulate miR-182-5p expression. Studies have demonstrated that ATG7 overexpression counteracts the autophagy-suppressing effects of CHRF silencing, indicating that CHRF promotes autophagy via the miR-182-5p/ATG7 pathway, ultimately exacerbating MIRI (Mo et al., 2021). Wang et al. used bioinformatics analysis and a dual-luciferase reporter assay to identify a binding site for miR-30a on both lncRNA AK088388 and Beclin-1 (Wang et al., 2019). In hypoxia-reoxygenation-treated cardiomyocytes, miR-30a expression was upregulated, whereas lncRNA AK088388, Beclin-1, and LC3-II expression were downregulated. Further experiments confirmed that lncRNA AK088388 functions as a ceRNA for miR-30a, leading to increased Beclin-1 and LC3-II expression, which in turn enhances autophagy and contributes to cardiomyocyte injury.

**FIGURE 3:**
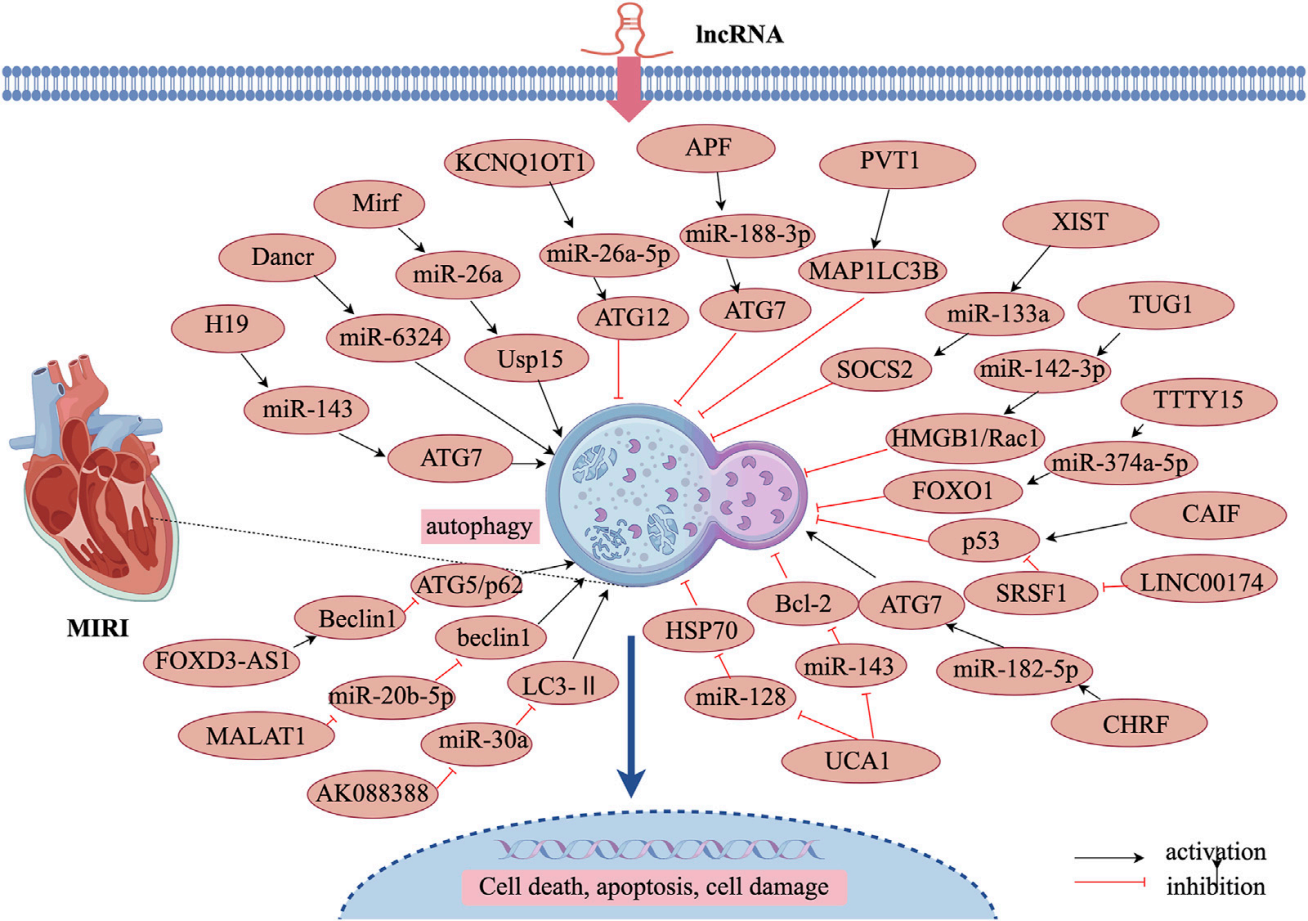
Mechanism of lncRNA-regulated autophagy in MIRI.

Oxygen-glucose deprivation/reoxygenation (OGD/R) is commonly used to establish an *in vitro* model of MIRI. Wang et al. (2019) demonstrated that lncRNA MALAT1 directly binds to miR-20b-5p and functions as a ceRNA, thereby regulating Beclin-1 expression. MALAT1 knockdown enhanced the inhibitory effect of miR-20b-5p on autophagy, reducing autophagy in OGD/R-injured cardiomyocytes. Conversely, lncRNA MALAT1 enhances Beclin-1-mediated autophagy by sponging miR-20b-5p, thereby aggravating OGD/R-induced cardiomyocyte injury. Additionally, FOXD3-AS1 expression was found to be increased in OGD/R-treated H9C2 cardiomyocytes. Overexpression of FOXD3-AS1 led to upregulation of LC3-II, Beclin-1, and ATG5, while downregulating p62 expression. Further studies revealed that FOXD3-AS1 overexpression activated the NF-κB/COX2/iNOS signaling pathway, thereby accelerating autophagy and worsening MIRI (Tong et al., 2019). These findings highlight FOXD3-AS1 as a potential regulator of ischemia-reperfusion injury through autophagy modulation, as illustrated in Figure 3 and Table 2.

**TABLE 2:** Role of lncRNA-regulated autophagy in MIRI.

lncRNA	Voice	Sample type	Target of action	Autophagy regulation	Mechanism of action	References
H19	adjust upwards	mice	miR-143/ATG7	Promoting autophagy	Mitigating the extent of MIRI	Lv et al. (2021a)
Dancr	adjust upwards	rats	miR-6324	Promoting autophagy	Restoration of endoplasmic reticulum homeostasis	Li et al. (2021a)
Mirf	adjust upwards	Cardiomyocytes and mice	miR-26a/Usp15	Promoting autophagy	Reduce infarct size and improve cardiac function	Liang et al. (2020)
KCNQ1OT1	adjust upwards	Mouse and cardiomyocytes	miR-26a-5p/ATG12	Inhibition of autophagy	Protecting cardiomyocytes	Li et al. (2021b)
APF	adjust upwards	Mouse cardiomyocytes	miR-188-3p/ATG7	Inhibition of autophagy	Slowing the progression of myocardial infarction	Wang et al. (2015)
PVT1	adjust upwards	miR-186/Beclin-1	MAP1LC3B	Inhibition of autophagy	LC3- II and Beclin-1 decreased expression levels and increased expression of p62	Ouyang et al. (2020)
XIST	adjust upwards	H9c2 cells and mouse myocardial tissue	miR-133a/SOCS2	Inhibition of autophagy	Promotes cellular vitality	Zeng et al. (2024)
TUG1	adjust upwards	H9c2 cells and mouse myocardial tissue	miR-142-3p/HMGB1/Rac1	Inhibition of autophagy	Improvement of myocardial injury	Li et al. (2019)
TTTY15	adjust upwards	mice	miR-374a-5p/FOXO1	Inhibition of autophagy	Reducing MIRI	Chen G. et al. (2021)
CAIF	adjust upwards	Mouse cardiomyocytes	p53/cardiac myosin	Inhibition of autophagy	Reduction of infarct size	Liu C. Y. et al. (2018)
LINC00174	demote	mice	SRSF1/P53/cardiac myosin	Inhibition of autophagy	Improvement of heart function	Su et al. (2021)
UCA1	adjust upwards	rats	miR-143/Bcl-2	Inhibition of autophagy	Protection of cardiac microvascular endothelial cells	Diao and Zhang (2021)
UCA1	demote	rats	miR-128/HSP70	Inhibition of autophagy	Reduced myocardial cell death and infarct size	Chen et al. (2018)
CHRF	adjust upwards	H9c2 cardiomyocytes	miR-182-5p/ATG7	Promoting autophagy	Exacerbation of MIRI	Mo et al. (2021)
AK088388	demote	HL-1	miR-30a/Beclin-1/LC3-II	Promoting autophagy	Aggravated MIRI	Wang et al. (2019)
MALAT1	demote	H9c2 cardiomyocytes	miR-20b-5p/beclin1	Promoting autophagy	Aggravated cardiomyocyte damage	Wang et al. (2019)
FOXD3-AS1	adjust upwards	H9c2 cardiomyocytes	LC3 II/Beclin1/ATG5/p62	Promoting autophagy	Aggravated MIRI	Tong et al. (2019)

### 5.3 CircRNA

#### 5.3.1 Overview of circRNAs

In 1976, Sanger et al. discovered the first circRNA in the cytoplasm of eukaryotic cells using an electron microscope. Due to the limited research methods available at the time, fewer circRNAs were identified, leading to the common belief that they were byproducts of aberrant splicing or what was referred to as “gene noise” (Chen Y. Q. et al., 2021). In 2012, Salzman (Salzman, 2016) from Stanford University discovered that circRNAs are abundant in the human body with the help of high-throughput sequencing technology. Since then, circRNAs have become a new generation of star molecules in the field of ncRNAs. CircRNAs are a class of stable and ubiquitous molecules in eukaryotic cells that lack a 5′-terminal cap and a 3′-terminal poly(A) tail. They are produced by back-splicing and are distributed in eukaryotic cells through various splicing mechanisms. The formation of circRNAs differs from the conventional splicing mode of linear RNA; instead, they are generated from precursor mRNA by back-splicing, where the downstream splicing acceptor is covalently linked to the upstream splicing donor, forming a circular structure. There are three different types of existing circRNAs: exonic circRNAs (exonic circRNAs, ecircRNAs), exon-intron circRNAs (exon-intron circRNAs, EIciRNAs), and intronic circRNAs (intronic circRNAs, ciRNAs) (Tang X. et al., 2021). CircRNAs have broad application prospects and have been found to be associated with various diseases, such as tumors, infectious diseases, hematological diseases, and autoimmune diseases. Their regulatory roles in the occurrence and development of human diseases have become a hot topic of research. Some circRNAs have also been found to be associated with the pathogenesis of pulmonary artery hypertension (Ali et al., 2022). The known mechanisms by which circRNAs function include acting as adsorbent sponges for miRNAs, interacting with proteins, regulating host proteins, functioning as super-enhancers, binding to DNA to form R-loops, and undergoing specific modifications. These diverse regulatory roles underscore the significance of circRNAs in cellular function and disease pathogenesis, making them promising targets for further investigation.

#### 5.3.2 CircRNA inhibition of autophagy attenuates MIRI

circRNAs can be degraded through autophagic processes, thereby regulating autophagy and contributing to the inhibition of MIRI progression (Si et al., 2019). PTEN-induced putative kinase 1 (Pink1), primarily expressed in the heart, muscle, and testis, plays a crucial role in autophagy regulation (Fan et al., 2020). circRNA ACR and circPAN3 have been identified as key modulators of autophagy in MIRI through Pink1 regulation. circRNA ACR, which is downregulated in hypoxia-reperfusion-induced cardiomyocytes, acts as an autophagy-regulating circular RNA by directly targeting DNA methyltransferase 3B (Dnmt3B) and activating Pink1 expression. This interaction prevents Dnmt3B-mediated DNA methylation at the Pink1 promoter, ensuring sustained Pink1 expression. Moreover, studies have demonstrated that Pink1 phosphorylation at serine 46 activates FAM65B, ultimately suppressing autophagy in cardiomyocytes (Zhou et al., 2019). Similarly, another study found that circPAN3 expression was significantly downregulated in a MIRI model, while circPAN3 overexpression markedly inhibited cardiomyocyte autophagy and alleviated myocardial injury. Mechanistically, miR-421 was identified as a downstream target of circPAN3, where it negatively regulates Pink1 expression. Furthermore, Pink1 downregulation counteracted the autophagy-inhibitory effects of circPAN3 overexpression, indicating that circPAN3 exerts its cardioprotective effects via the circPAN3–miR-421–Pink1 axis (Zhang et al., 2021). These findings highlight the therapeutic potential of circRNAs in MIRI through autophagy regulation, providing novel insights into potential treatment strategies for MIRI.

Another study demonstrated that circRNA_101237 functions as a ceRNA for let-7a-5p, thereby regulating insulin-like growth factor 2 mRNA-binding protein 3 (IGF2BP3)-dependent autophagy. let-7a-5p inhibits cardiomyocyte death by targeting IGF2BP3 and suppressing autophagy. Therefore, downregulation of circRNA_101237 leads to decreased IGF2BP3 expression, which consequently inhibits hypoxia/reoxygenation-induced autophagy in primary cardiomyocytes (Gan et al., 2020). Similarly, circZNF292 was significantly upregulated in oxygen-glucose-deprived cardiomyocytes. BCL2/BNIP3, a key regulator of autophagy and apoptosis, is closely associated with the Wnt/β-catenin and mTOR signaling pathways (Ren et al., 2019). Given its function, circZNF292 inhibits cardiomyocyte death by activating the Wnt/β-catenin and mTOR pathways, which target BNIP3 to suppress autophagy, thereby attenuating oxygen-glucose deprivation-induced ischemic injury in H9c2 cells. Furthermore, the knockdown of circDGKZ suppressed NLRP3 and cleaved-caspase1 expression while enhancing LC3-II/I levels. However, additional knockdown of ATG5 led to upregulated NLRP3 and cleaved-caspase1 expression while reducing LC3-II/I levels, confirming that circDGKZ knockdown inhibits autophagy by regulating miR-345-5p-mediated autophagy in cardiomyocytes, ultimately improving MIRI outcomes (Li et al., 2024).

#### 5.3.3 CircRNA inhibition of autophagy aggravates MIRI

**FIGURE 4:**
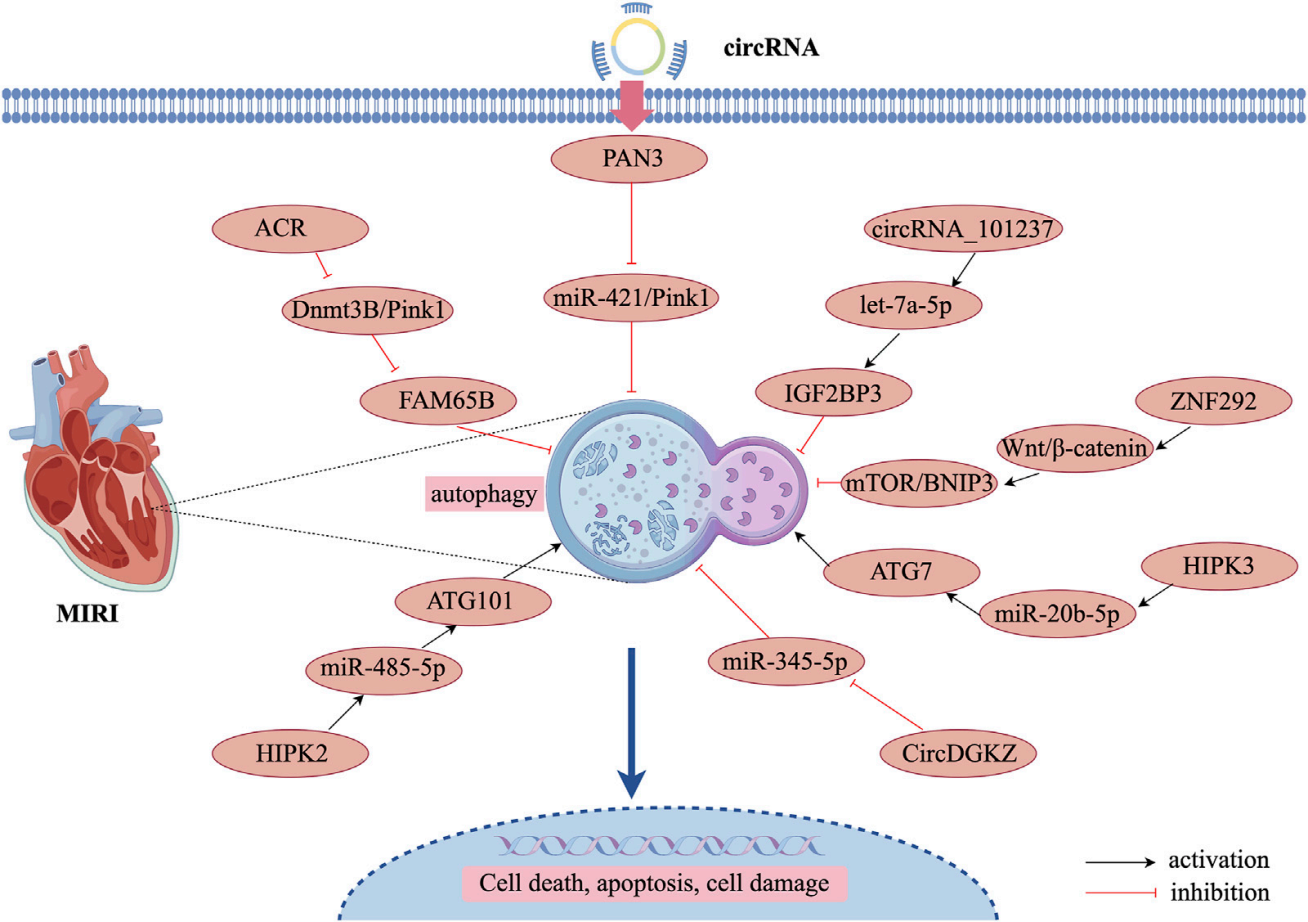
Mechanism of circRNA-regulated autophagy in MIRI.

The activation of autophagy following MIRI is closely associated with the upregulation of autophagy-related genes (ATG7 and ATG101). During MIRI and cardiomyocyte hypoxia/reoxygenation injury, the expression of circHIPK3 is significantly upregulated, contributing to enhanced autophagy in hypoxia/reoxygenation-treated cardiomyocytes. circHIPK3 acts as a molecular sponge for miR-20b-5p, which directly targets ATG7. Consequently, circHIPK3 promotes cardiomyocyte autophagy and cell death by suppressing miR-20b-5p and increasing ATG7 expression (Qiu et al., 2021). Furthermore, ATG101 knockdown enhances proliferation and limits cell death by attenuating autophagy in H_2_O_2_-injured neonatal mouse cardiomyocytes. Additionally, circHIPK2 positively regulates ATG101 expression by acting as a sponge for miR-485-5p (Zhou et al., 2020). In summary, circHIPK2 promotes H_2_O_2_-induced autophagy and cardiomyocyte death via the miR-485-5p/ATG101 pathway, thereby exacerbating myocardial oxidative injury. miR-345-5p directly regulates human growth arrest-specific protein 6 and high glucose-induced pro-inflammatory cytokine release and cardiomyocyte apoptosis (Ma et al., 2020). circDGKZ directly interacted with miR-345-5p, and knockdown of circDGKZ reduced miR-345- 5p adsorption, thereby inhibiting TLR4 signalling, which in turn promotes autophagy-induced NLRP3 degradation, inhibits cardiomyocyte focal death, and ultimately attenuates MIRI (Li et al., 2024). The specific regulatory mechanisms of these circRNAs are illustrated in Figure 4 and Table 3.

**TABLE 3:** Role of circRNA-regulated autophagy in MIRI.

circRNA	Voice	Sample type	Target of action	Autophagy regulation	Mechanism of action	References
ACR	demote	mice	Dnmt3B/Pink1/FAM65B	Inhibition of autophagy	Protecting the heart from ischemia-reperfusion injury	Zhou et al. (2019)
PAN3	demote	mice	miR-421/Pink1	Inhibition of autophagy	Reduction of myocardial infarct size	Zhang et al. (2021)
circRNA_101237	adjust upwards	mice	let-7a-5p/IGF2BP3	Inhibition of autophagy	Reduced cardiomyocyte death	Gan et al. (2020)
ZNF292	adjust upwards	H9c2 cardiomyocytes	Wnt/β-catenin/mTOR/BNIP3	Inhibition of autophagy	Reducing ischemic injury in cardiac myocytes	Ren et al. (2019)
HIPK3	adjust upwards	Mouse cardiomyocytes	miR-20b-5p/ATG7	Promoting autophagy	Promotion of apoptotic cell death in cardiomyocytes	Qiu et al. (2021)
HIPK2	adjust upwards	Mouse cardiomyocytes	miR-485-5p/ATG101	Promoting autophagy	Promotion of apoptotic cell death in cardiomyocytes	Zhou et al. (2020)
CircDGKZ	demote	Rat cardiomyocytes	miR-345-5p	Inhibition of autophagy	Reducing MIRI	Li et al. (2024)

## 6 Conclusion

With the continuous development of bioinformatics and gene sequencing technology, it is found that more and more ncRNAs with biological functions are involved in the occurrence and development of MIRI, such as miRNAs mainly affecting the key pathways of ischemic preconditioning or ischemic postconditioning, lncRNAs affecting MIRI mainly by affecting miRNAs or directly acting on mRNAs through the high-level spatial structure, and mitochondrial function is its main mediating site, while circRNA plays its regulatory role mainly through the sponge adsorption of miRNAs and thus affects the target genes downstream of miRNAs, and lncRNAs, miRNAs, and circRNAs can regulate autophagy and thus alleviate or exacerbate MIRI by promoting, inhibiting, and promoting/inhibiting it, *etc.* Therefore, this report summarizes the autophagic regulation mechanism and the pathways involved in MIRI-related signaling pathways, and summarizes and sorts out the mechanism of non-coding RNA-regulated autophagy in MIRI, which provides a new potential target for the treatment of MIRI.

Our study determined by inductive analysis that during the ischemic phase of myocardial ischemia, autophagy is activated due to energy crisis and oxidative stress in cardiomyocytes, thereby maintaining the structure and function of cardiomyocytes and protecting the physiological function of the heart. During the reperfusion phase, autophagy is overactivated, leading to cardiomyocyte autophagic death and tissue damage. Therefore, moderate levels of autophagy are involved in myocardial reperfusion injury and cardiomyocyte hypoxia-reoxygenation processes and maintain cardiomyocyte homeostasis. In addition, autophagy exerts a regulatory effect on MIRI by regulating signaling pathways such as PI3K/Akt/mTOR, AMPK/mTOR, SIRT3 and BNIP3. Secondly, miRNAs participate in the process of MIRI by promoting autophagy, which inhibits cardiomyocyte death and protects ischemic-hypoxic-injured cardiomyocytes, while some miRNAs can protect damaged cardiomyocytes by inhibiting the over-activation of autophagy, and some miRNAs can exacerbate MIRI by promoting or inhibiting autophagy, which can have a detrimental impact on the heart. lncRNAs are able to promote autophagy and thereby exert a regulatory effect on MIRI during MIRI. promote autophagy during MIRI and thus exert a protective effect on cardiomyocytes. part of lncRNAs can inhibit the over-activation of autophagy by regulating autophagy-related genes and thus maintain their cellular homeostasis, and the other part of lncRNAs are upregulated after MIRI and adversely affect the heart by repressing the expression of miRNAs and enhancing the expression of autophagy-related target genes. circRNAs are subject to cleaved by autophagic degradation and regulate autophagy accordingly, thus inhibiting the developmental process of MIRI, and part of cricRNAs inhibit autophagy to aggravate MIRI.

However, current studies have focused on the discovery and screening of ncRNAs and their value as biomarkers, while the specific mechanisms have not been fully elucidated. First, there is a lack of studies on epigenetic modifications and interactions with RNA-binding proteins, such as lncRNAs acting as molecular bridges mediating the co-localization of RNA-binding proteins with epimodifying enzymes, which synergistically regulate the expression targets of genes related to apoptosis, mitochondrial metabolism, and fibrosis in MIRI cardiomyocytes. Second, in the study of noncoding RNA intervention in autophagy regulation of MIRI, there is significant heterogeneity in the results of different cell models (e.g., H9c2 *versus* AC16) and pathological stages (e.g., ischemia *versus* reperfusion). Third, the *in vivo* delivery efficiency, off-target effects, and druggability challenges of ncRNAs as biomarkers or therapeutic targets have not been sufficiently explored, and there is a lack of studies on the regulatory mechanisms of MSC-derived exosomes and nanoparticle-based non-coding RNA delivery systems in MIRI. Fourth, there is a lack of ncRNA-based therapies such as the miR-92a inhibitor MRG-110 for MIRI in clinical trials. Fifth, the current methods used to assess autophagy levels mainly include the expression of related genes, proteins, and signaling pathways, and there is a lack of application of emerging technologies such as autophagy reporter gene systems. Sixth, ncRNA-based interventions need to optimize the targeted delivery system to reduce off-targeting, modification to reduce immunogenicity, long-term safety needs to validate nucleic acid persistence, genomic integration risk, and vector toxicity, and the lack of tracking data constrains clinical translation. Seventh, the existing studies are dominated by single lncRNA, miRNA and circRNA studies, and lack of studies on crosstalk between miRNA-lncRNA-circRNA interaction networks. Eighth, because the regulation of autophagy by ncRNAs usually involves multi-targets and multi-pathways and the risk of invasive operation in obtaining myocardial tissues for clinical research, most of the studies in this field are limited to animal models, and its real application in the clinic still requires long-term exploration.

Therefore, further multi-center and large-scale clinical trials with multi-pathway crosstalk and multi-model intervention studies must be conducted in the future, combined with single-cell sequencing data in order to reveal the cell-type specific role of ncRNAs in autophagy, as well as to construct an interaction network connecting ncRNAs, autophagy, inflammation, iron death and pyroptosis in MIRI, and to explore the specific pathways of ncRNAs regulating autophagy in MIRI. the specific pathways of action of non-coding RNA-regulated autophagy in MIRI, which is expected to provide a new direction for the clinical treatment of MIRI.
